# Características clínicas e imagenológicas del cementoma gigantiforme familiar. Ana revisión de la literatura

**DOI:** 10.21142/2523-2754-0903-2021-073

**Published:** 2021-10-06

**Authors:** Grizel Karem Rodríguez-Cuentas

**Affiliations:** 1 Facultad de Odontología, Universidad de Aquino Bolivia. Oruro, Bolivia. griss_rc7@hotmail.com Universidad de Aquino Bolivia Facultad de Odontología Universidad de Aquino Bolivia Oruro Bolivia griss_rc7@hotmail.com

**Keywords:** cementoma gigantiforme familiar, lesión fibro-ósea, tomografía computarizada de haz cónico, Familial Gigantiform Cementoma, fibro-osseous injury, Computed Tomography Cone Beam

## Abstract

El cementoma gigantiforme familiar es una lesión fibro-ósea benigna poco común, que sigue un patrón de herencia autosómico dominante. Se presenta durante la infancia y se limita a los huesos de la cara, especialmente la mandíbula. Presenta crecimiento rápido e indoloro, y se expande considerablemente con el paso de los años. Está considerada entre los siete trastornos que afectan la fisonomía del esqueleto craneofacial. Radiográficamente, se produce en tres etapas de maduración, al igual que las displasias óseas: radiolúcida, mixta y radiopaca, y se describe como una masa lobular mixta, bien delimitada, que puede aparecer en ambos maxilares y provocar la expansión de corticales óseas vestibular y palatina/lingual, el desplazamiento y la retención de piezas dentarias. Este artículo tuvo el objetivo de realizar una revisión de la literatura más relevante para identificar las características clínicas, radiográficas e histopatológicas del cementoma gigantiforme familiar en los maxilares, además de mostrar las herramientas imagenológicas útiles para el diagnóstico y seguimiento de la lesión.

## Abstract (en)

Familial gigantiform cementoma (FGC) is a rare benign fibro-cementum lesion, which follows an autosomal dominant inheritance pattern and presents during childhood. It is limited to the bones of the face, with a predilection for the jaw, is fast growing and painless and expands considerably over time. It is considered among the seven disorders that affect the physiognomy of the craniofacial skeleton. Radiographically, FGC occurs in three stages of maturation similar to bone dysplasia, being radiolucent, mixed and radiopaque and is described as a mixed lobular well delimited mass, which can occur in both maxillae, causing expansion of the buccal and palatal / lingual bone cortices. displacement and retention of teeth. The aim of this study was to perform a review of the literature to identify the clinical, radiographic and histopathological characteristics of FGC in the jaws and describe the imaging tools that are useful for the diagnosis and follow-up of this lesion.

## INTRODUCCIÓN

El cementoma gigantiforme familiar (CGF) es una lesión fibro-ósea benigna poco común, que sigue un patrón de herencia autosómico dominante ^1^^-^^3^. Se ha visto en familias de distintas partes del mundo, como Italia, Estados Unidos, Corea y Filipinas, y predomina en pacientes de raza negra, blanca y de Asia oriental ^2^^,^^4^.

En 1971, la Organización Mundial de la Salud (OMS) clasificó al CGF en el subgrupo de cementomas. Norberg (1930), Agazzi y Belloni (1941) serían los que comenzaron a estudiar esta lesión. Por otra parte, Yung et al. (1989) también reportan las características clínicas de la lesión ^2^^,^^5^.

La OMS, en el 2005, la clasificó como displasia ósea y, en el 2017, como lesión fibro-ósea, según edad, sexo, localización de la lesión y características clínicas, imagenológicas e histopatológicas. El CGF está clasificado entre las lesiones fibro-óseas y condromatosas que incluyen al fibroma osificante, la displasia fibrosa, la displasia cemento-ósea y el osteocondroma ^6^^,^^7^. Se caracteriza por ser una lesión expansiva, que afecta los cuatro cuadrantes de los maxilares, con predilección de la mandíbula ^8^^-^^10^. Está incluido entre los siete trastornos que afectan la fisonomía del esqueleto craneofacial, acompañado por la displasia fibrosa, la osteogénesis imperfecta, la osteopetrosis, la displasia cleidocraneal, el querubismo y el síndrome de Gardner ^11^. La remoción quirúrgica completa de la lesión es el tratamiento más eficaz para evitar futuras recidivas; sin embargo, la rápida proliferación y reaparición de la lesión hace más difícil su eliminación total ^2^^,^^8^^,^^11^. Se debe realizar un estudio minucioso de la historia clínica familiar, el cual, en conjunto con los exámenes imagenológicos e histopatológicos de la lesión, puede contribuir a su diagnóstico definitivo ^3^^,^^12^.

El CGF, al ser poco frecuente, no ha sido investigado por completo y existen pocas revisiones en la literatura sobre pacientes con esta patología. El presente trabajo de investigación tiene como objetivo realizar una revisión de la literatura de las características clínicas, imagenológicas e histopatológicas del cementoma gigantiforme familiar, para poder diferenciarlo de otras lesiones similares, además de señalar las herramientas imagenológicas útiles para el odontólogo en su seguimiento y tratamiento.

## MATERIALES Y MÉTODOS

Se realizó una búsqueda bibliográfica en las principales bases de datos de la literatura científica internacional sobre ciencias de la salud: Medline, a través de PubMed, Scopus y SciELO, utilizando las palabras clave “cementoma gigantiforme”, “cementoma gigantiforme familiar”, “lesiones fibro-óseas” y “tomografía computarizada de haz cónico”. Se incluyeron artículos de revisión publicados entre el año 2000 y junio del 2020. 

## CARACTERÍSTICAS CLINICAS, IMAGENOLÓGICAS E HISTOPATOLÓGICAS DEL CEMENTOMA GIGANTIFORME FAMILIAR

El cementoma gigantiforme familiar fue descrito como una forma rara de presentación de fibroma osificante de etiología desconocida, que involucra zonas de hueso alveolar y de tipo familiar, aunque también existen algunos casos reportados sin antecedentes familiares ^2^. Las características clínicas de pacientes diagnosticados con CGF en la literatura indican su aparición exclusiva en los maxilares, durante la infancia, entre la primera y segunda década de vida. Es de crecimiento rápido e indoloro, se expande considerablemente con el paso de los años, abarca los cuatro cuadrantes y provoca deformidad facial en edad adulta. Finalmente, este crecimiento progresivo de la lesión se detiene en la quinta década de vida, con o sin tratamiento de la patología ^2^^,^^3^^,^^13^.

Existen artículos que mencionan las fracturas en extremidades inferiores en pacientes con CGF ^1^^,^^2^^,^^4^^,^^11^. Uno de los primeros estudios describió un caso heredado que afectó a cuatro generaciones, y la primera familia diagnosticada con CGF estuvo en China, con un total de 13 portadores. Apareció entre los 11 y 13 años, luego las lesiones crecieron y se expandieron considerablemente entre los 14 y 16 años, con predominio en la región anterior de la mandíbula. Algunos presentaron varias fracturas en extremidades inferiores por traumatismos menores, y el fémur fue el hueso más afectado. Sus niveles de fosfatasa alcalina estaban elevados y los niveles de calcio, bajos. Hubo una disminución en el crecimiento de las lesiones entre los 18 y 20 años, y no se presentaron nuevamente fracturas en huesos largos ^2^.

Revisiones de la literatura describen radiográficamente al CGF como múltiples masas mixtas, bien delimitadas, que se presentan en maxilar superior e inferior, y que en la mayoría de casos empieza en la región anterior de la mandíbula. En imágenes 2D, se observan múltiples áreas radiolúcidas y radiopacas, bien delimitadas y lobulares, rodeadas por un halo radiolúcido, que pueden ser muy expansivas y logran así pasar la línea media, con lo que abarcan ambos maxilares. Además, se puede observar la retención y desorganización de piezas dentarias ^2^^,^^15^^,^^16^.

La evolución del CGF se describe, en la literatura, en tres etapas de maduración, al igual que las displasias óseas: radiolúcida, mixta y radiopaca. En la primera, se presentan imágenes radiolúcidas pequeñas localizadas en el hueso alveolar. Se caracteriza por estar formada por tejido celular sin presencia de calcificaciones. Posteriormente, en la maduración de la lesión, pasa a una etapa mixta con imágenes radiolúcidas y radiopacas, que reemplazan el hueso normal, para luego pasar a una etapa más madura, donde se observan masas radiopacas lobulares y de gran tamaño con características expansivas, lo que provoca expansión de corticales óseas vestibular y palatina/lingual, desplazamiento y retención de piezas dentarias ^3^^,^^11^^,^^17^. Estas masas radiopacas presentan un alto riesgo de infectarse y producir una osteomielitis ^5^.

Estudios realizados mediante el uso de la tomografía evidencian que estas lesiones no se relacionan con superficies radiculares de piezas dentarias y que la zona más afectada suele ser la de premolares y molares ^3^^,^^9^^,^^18^^,^^19^. Por esta razón, varios autores sugieren quitar el nombre de “cementoma” de la terminología ^17^^,^^20^.

Histológicamente, el CGF es descrito como fibromas cemento osificantes de hueso inmaduro y masas calcificadas, que pueden tener una composición variada de tejido fibroso y calcificado ^14^. En un reporte de caso, se demostró que la toma de la muestra para la biopsia no es fácil, al ser una lesión de consistencia dura y hemorrágica, por lo tanto, no se puede separar del hueso de manera sencilla ^21^.

Luego del análisis de la literatura, para llegar a un diagnóstico definitivo, es primordial realizar una buena anamnesis que, en conjunto con los exámenes imagenológicos e histopatológicos, nos permitirá diferenciarla de otras lesiones patológicas similares. 

## EXÁMENES IMAGENOLÓGICOS DE UTILIDAD PARA LA IDENTIFICACIÓN DEL CEMENTOMA GIGANTIFORME FAMILIAR

Para la evaluación de las lesiones fibro-óseas en maxilares, incluyendo el CGF, es necesario conocer qué estudios imagenológicos son fundamentales para llegar al diagnóstico de la patología. Entre las herramientas imagenológicas están la radiografía 2D, la tomografía computarizada de haz cónico, la resonancia magnética y la gammagrafía ^2^^,^^16^.

El estudio imagenológico más simple y de bajo costo es la radiografía panorámica, que fue introducida en 1960 ^22^. Generalmente, es el primer estudio para la evaluación de la lesión en dos dimensiones y permite la visualización temprana de características radiográficas de la lesión, en algunos casos por hallazgos que permiten identificarlo de manera oportuna, observándose múltiples lesiones radiopacas y radiolúcidas bien circunscritas y multiloculares que comprometen ambos maxilares. Además, permite observar la alteración en la posición de las piezas dentarias, incluyendo las retenidas dentro de las lesiones ^3^. Sin embargo, este estudio presenta ciertas limitaciones debido a que es una imagen 2D, lo que no permite evaluar la lesión en su totalidad, pues presenta superposición de estructuras, desenfoque de la imagen y magnificación, lo que limita la realización de mediciones ^22^.

Antiguamente, no se contaba con estudios de última generación como la tomografía computarizada de haz cónico (TCHC), implementada en 1998, exclusiva-mente para uso dental. Ésta permite obtener imágenes tomográficas y reconstrucciones volumétricas tridi-mensionales de las estructuras orales y maxilofaciales. ^23^^,^^24^. A diferencia de la tomografía computarizada (TC) convencional, la TCHC utiliza una dosis mínima de radiación, tiene más precisión en la adquisición de imágenes con una mejor resolución. Hasta ahora es una herramienta muy útil y valiosa, con múltiples aplicaciones en la odontología ^24^^,^^25^. 

La evaluación del CGF mediante esta herramienta permite su evaluación en tres dimensiones, utilizando menor dosis de radiación y obteniendo, además, imágenes con alta resolución en vistas axiales, coronales y sagitales, así como reconstrucciones en tres dimensiones, para determinar la extensión de la lesión, el tamaño y el compromiso de las estructuras anatómicas adyacentes, facilitando la evaluación, el diagnóstico y el seguimiento de esta patología ^22^^,^^26^^-^^28^. La imagen muestra lesiones heterogéneas múltiples y bilaterales, rodeadas de áreas hipodensas que provocan la expansión y el adelgazamiento de las corticales óseas vestibular y palatina/lingual, mayormente, la cortical ósea vestibular ^29^. Así mismo, se observa la retención y el desplazamiento de piezas dentarias a diferentes direcciones ^13^.

La resonancia magnética y la gammagrafía son herramientas de diagnóstico que complementan el estudio y permiten observar también este tipo de patología. Aunque no se encontraron estudios con resonancia magnética reportados en la literatura, es considerado un examen de utilidad cuando la lesión compromete tejido blando adyacente.

La gammagrafía es de mayor utilidad en una fase temprana de la lesión. Aunque son pocos los casos de CGF evaluados con gammagrafía, los realizados muestran alta intensidad de captación de radiotrazadores que se observan en este tipo de patología en los maxilares ^8^^,^^21^^,^^30^. La medicina nuclear tiene distintos isótopos para aplicaciones en el diagnóstico y uno de los métodos más utilizados para localizar anomalías en el sistema esquelético es la gammagrafía ósea MDP marcada con 99mTc ^19^.

## DIAGNÓSTICOS DIFERENCIALES DEL CEMENTOMA GIGANTIFORME Y EL APORTE DE LA IMAGENOLOGÍA PARA LOGRAR DIFERENCIARLOS

Entre los diagnósticos diferenciales del CGF están las displasias óseas (focal, periapical y florida), fibroma osificante, cementoblastoma benigno, osteomielitis esclerosante crónica difusa ^9^^,^^13^^,^^15^.

La displasia ósea periapical (DOP) es más frecuente en mujeres, entre los 30 y 50 años, y apical a incisivos inferiores. La mucosa oral no se ve afectada y suele ser observada mediante hallazgo radiográfico. Radiográficamente, se presenta en una etapa inicial como una imagen radiolúcida a nivel de los ápices de piezas dentarias con vitalidad pulpar; no se observa reabsorción radicular ni desplazamientos dentarios. Son asintomáticos y se pueden confundir con una lesión apical, para lo cual se realiza un tratamiento endodóntico o quirúrgico de la pieza. Posteriormente, esta lesión incrementa su mineralización a una imagen más radiopaca, de límites definidos e irregulares, rodeada por un halo radiolúcido y un área radiopaca de hueso denso en la periferia de la lesión ^31^. Esta lesión no es expansiva, pero rara vez puede medir más de un centímetro y puede expandir levemente las corticales óseas, observadas en imágenes 2D, por lo que se puede diagnosticar sin necesidad de recurrir a un examen histopatológico ^32^.

La displasia ósea focal (DOF) predomina en mujeres de raza negra, en una edad promedio de 38 a 41 años ^33^. Se caracteriza por ser una lesión solitaria y se localiza más frecuentemente a nivel de molares inferiores. Generalmente, es de hallazgo radiográfico y no presenta sintomatología. Radiográficamente, se observa igualmente en una primera etapa una imagen radiolúcida de límites definidos con o sin área esclerótica en la periferie de la lesión. Posteriormente, cambia de forma progresiva a una radiopacidad ^31^ que no pasa los dos centímetros en el diámetro. Se requiere de un examen histopatológico para determinar su diagnóstico.

La displasia cemento-ósea florida (DCOFl) es una lesión fibro-ósea multifocal benigna de etiología desconocida, que afecta a mujeres de raza negra, entre la cuarta y quinta década de vida; es asintomática y, generalmente, son hallazgos radiográficos. Se presenta bilateralmente y puede afectar los cuatro cuadrantes en los maxilares ^34^^,^^36^. Radiográficamente, se presentan en tres etapas: radiolúcida, mixta y radiopaca, mayormente en zona de premolares y molares. Se observan múltiples masas radiopacas rodeadas de áreas radiolúcidas, que se unen entre sí y forman masas con mayor radiopacidad y lobulares, presentándose más intensas en las crestas alveolares. Pueden alcanzar un gran tamaño, que compromete en algunos casos la mucosa oral y hace que el tejido óseo sea más propenso a infecciones, lo que podría desencadenar en una osteomielitis crónica supurativa ^16^^,^^37^^,^^39^. La DCOFl familiar no es tan frecuente, con pocos casos reportados en la literatura, pero se demostró que podría ser hereditaria de forma autosómica dominante, con expresión fenotípica variable ^9^^,^^39^^,^^40^.

Histopatológicamente, las tres displasias óseas son similares. En la primera etapa están formadas por tejido conectivo celular fibroso, vascularizado y con presencia de hueso inmaduro, osteoblastos y calcificaciones ^33^. El CGF está compuesto por cantidades de masas calcificadas, con tejido fibroso y mineralizado. Todos los estudios realizados y evaluados debidamente y con conocimiento de las características de esta patología y sus manifestaciones clínicas, radiográficas e histopatológicas, nos pueden llevar a su diagnóstico definitivo ^5^.

El fibroma osificante (FO) es una neoplasia benigna de la mandíbula, que se presenta entre la tercera y cuarta década de vida, y puede llegar a expandirse considerablemente sin presencia de dolor. Tiene características radiográficas similares al CGF, pero rara vez involucra los cuatro cuadrantes. En su etapa inicial, se observa una imagen radiolúcida unilocular en el tejido óseo alveolar; posteriormente, aparecen zonas radiopacas en su interior y llega a ser netamente radiopaca en la etapa final. Se expande de forma centrífuga hacia el exterior y, debido a su expansión, provoca deformidad facial y desplazamiento de piezas dentarias, con posible resorción radicular, manteniendo la vitalidad pulpar ^41^^,^^42^.

Revisiones de la literatura mencionan al CGF como una presentación clínica, radiológica e histopatológica muy parecida a la DCOFl. Ambas lesiones pasan por tres etapas de maduración, se presentan bilateralmente y expanden corticales, pero el CGF, además, se caracteriza por tener una tendencia familiar, no tiene predilección de sexo y raza, la aparición de la lesión se da a edad temprana (generalmente durante la infancia), es de crecimiento rápido y provoca deformidad facial ^2^^,^^5^. Radiográficamente, el CGF se caracteriza por ser agresivo, al expandirse rápidamente en ambos maxilares, lo que compromete el reborde óseo alveolar y basal mandibular, y ocasiona deformidad facial y maloclusión, lo cual no sería característico de la DCOFl ^8^^,^^31^.

El cementoblastoma benigno (CB) es una lesión odontogénica benigna, que se presenta más en la región de primeros molares inferiores. Radiográficamente, se aprecia una imagen radiopaca unida a la raíz de la pieza dentaria comprometida, rodeada por un margen radiolúcido bien delimitado ^13^. Es difícil diferenciar esta lesión con CGF, pues ambas pueden aparecer en zona de molares como masas radiopacas, y están rodeadas por un halo radiolúcido y una masa radiopaca en la periferie. 

La osteomielitis esclerosante difusa (OED) es una lesión común que puede causar dolor e inflamación. Se presenta en adultos sin predilección de sexo y se localiza en la mandíbula, mayormente unilateral, comprometiendo cuerpo, ángulo, rama ascendente y cóndilo mandibular. Radiográficamente, se observa una lesión radiolúcida con esclerosis difusa o radiopacidad en espacios medulares adyacentes a piezas dentarias ^43^^,^^44^.

Luego del análisis de la literatura, es importante mencionar que, cuando el odontólogo se enfrenta a esta patología, podría tener cierta dificultad para determinar el tipo de lesión, pues existen otras muy similares radiológicamente y no se puede determinar de manera exacta; por lo tanto, es indispensable complementar con un estudio histopatológico. 

## DISCUSIÓN

El cementoma gigantiforme familiar, al ser una patología poco frecuente, no ha sido investigado a fondo, lo cual ha dificultado el plan de tratamiento y rehabilitación oral en los casos reportados de la literatura. Para su identificación, se debe realizar una evaluación minuciosa, a fin de reconocer las características clínicas e imagenológicas, y así descartar los posibles diagnósticos diferenciales, además de correlacionarlo con un estudio histopatológico para un diagnóstico definitivo.

El CGF es de etiología desconocida, su aparición es exclusiva en maxilares durante la infancia, presenta crecimiento rápido e indoloro, y se expande rápidamente provocando deformidad facial. También se debe tomar muy en cuenta que los pacientes con CGF son propensos a tener fracturas de huesos largos en las extremidades inferiores ^2^^,^^4^^,^^13^. El seguimiento de la primera familia en China diagnosticada con CGF ^2^ permitió afirmar que la mayoría de los pacientes con CGF evaluados habían tenido al menos una historia de fractura en huesos largos, y todos coincidieron en que las fracturas habían ocurrido en la fase 2 de la lesión, que fue entre los 14 y 16 años, tiempo durante el cual esta se había expandido más rápidamente, en comparación con las fases 1 y 3. Esto lleva a la conclusión de que el CGF podría estar asociado a la disminución de calcio, razón por la cual se producen las fracturas en las extremidades inferiores y, al mismo tiempo, se expande. También se evidenció que, en la etapa más tardía de la lesión, en la que no se presentaron fracturas, no se expandía la lesión. En cuanto a la selección del examen imagenológico para CGF, las radiografías bidimensionales pueden ser de gran ayuda, pero no se puede realizar una completa evaluación de la lesión debido a la superposición de estructuras y la limitación de imagen 2D ^22^. 

La TCHC es hasta ahora una técnica imagenológica con un gran aporte al diagnóstico, la planificación del tratamiento y el seguimiento de una determinada patología, con un alto nivel de resolución y precisión, y una mínima dosis de radiación en comparación con la tomografía computarizada (TC) ^27^. Para la evaluación de CGF, es preferible el estudio y seguimiento mediante la TCHC, al ser una patología que aparece en la infancia, por lo que será mejor utilizar una baja dosis de radiación. Además, esto permitirá evaluar totalmente la lesión, al permitir la visualización por planos sagital, axial y coronal, y reconstrucciones 3D, para de este modo analizar completamente la lesión, su localización, contornos, tamaño y relación con estructuras anatómicas adyacentes, así como corticales y valoración del tejido óseo. Todo esto es muy importante para garantizar la escisión quirúrgica completa de la lesión, lo cual es recomendable por su alta recurrencia e infección en casos de escisión parcial. Revisiones de la literatura revelan que pacientes intervenidos quirúrgicamente con eliminación parcial de la lesión resultan en recurrencia y crecimiento de la lesión. 

El diagnóstico diferencial del CGF con otras lesiones fibro-óseas es complicado y aún está en discusión, pues existen características radiográficas e histológicas similares. Por esta razón, también es necesaria una evaluación clínica, acompañada de exámenes auxiliares radiológicos e histopatológicos minuciosos, que permitan diferenciarla de otro tipo de lesiones y así obtener un diagnóstico definitivo. Debido al poco número de casos reportados en la literatura, no se ha establecido formalmente un tratamiento a seguir para la rehabilitación esperada, pero se sugiere realizar un análisis de los niveles de calcio, fósforo y fosfatasa alcalina. Además, se aconseja no realizar procedimientos quirúrgicos incompletos ni segundas cirugías, debido a la alta recurrencia con crecimiento más rápido de las lesiones; lo recomendable es hacer un seguimiento, para vigilar que no existan factores que puedan dar lugar a una infección.

Aunque se presentaron dificultades en la búsqueda de información, debido a los pocos casos reportados en la literatura, se revisaron también los artículos no relacionados directamente con el cementoma gigantiforme familiar, y se logró reunir literatura importante para su estudio. 

Se recomienda seguir con la investigación de esta patología, debido a que no se ha dado total importancia a su estudio. Además, el nombre de cementoma gigantiforme familiar dado a esta patología viene siendo discutido por varios autores, pues se ha observado, mediante estudios imagenológicos, que no existe relación entre el cemento radicular y las lesiones, por lo cual no sería prudente hablar de “cementoma”. También se pone en duda el término “gigantiforme”, pues solo existe una expansión en la etapa media de la lesión y luego se detiene en su etapa madura. Lo mismo ocurre con el término “familiar”, pues se han reportado casos sin antecedentes familiares. 

## CONCLUSIONES

El CGF es un tumor cuya descripción no está del todo clara, debido a que, por su rara aparición, no existe mucha información ni estudios suficientes al respecto. La tomografía computarizada de haz cónico es el estudio de mayor utilidad para su análisis imagenológico y posible diagnóstico presuntivo; además, ayuda en el plan de tratamiento y seguimiento de esta patología, porque gracias a las vistas sagitales, axiales, coronales y la reconstrucción 3D que nos ofrece, se puede evaluar la extensión de la lesión y detectar posibles recurrencias posquirúrgicas. El odontólogo, en el ejercicio de su profesión, se enfrenta con la probabilidad de encontrar este tipo de lesiones en los maxilares y debe estar preparado para identificarlas; asimismo, debe correlacionar las características radiográficas e histológicas con los datos clínicos para llegar a un diagnóstico definitivo de CGF, pues existen otras lesiones similares, pero que se manejan de distinta manera y tienen un protocolo de tratamiento diferente, de acuerdo con el comportamiento de la lesión. 

## Fuente de financiamiento:

Autofinanciado
